# Success and High Predictability of Intraorally Welded Titanium Bar in the Immediate Loading Implants

**DOI:** 10.1155/2014/215378

**Published:** 2014-05-22

**Authors:** Vaniel Fogli, Michele Camerini, Dorina Lauritano, Francesco Carinci

**Affiliations:** ^1^Private Practicing, Via Mariotti Franceschino n° 8, 48022 Ravenna, Italy; ^2^Neuroscience and Biotechnology Department, University of Milano-Bicocca, Via Cadore n° 48, 20052 Monza, Italy; ^3^Department of Morphology, Surgery and Experimental Medicine, Maxillofacial School, University of Ferrara, Via Fossato di Mortara n° 52, Ferrara, Italy

## Abstract

The implants failure may be caused by micromotion and stress exerted on implants during the phase of bone healing. This concept is especially true in case of implants placed in atrophic ridges. So the primary stabilization and fixation of implants are an important goal that can also allow immediate loading and oral rehabilitation on the same day of surgery. This goal may be achieved thanks to the technique of welding titanium bars on implant abutments. In fact, the procedure can be performed directly in the mouth eliminating possibility of errors or distortions due to impression. This paper describes a case report and the most recent data about long-term success and high predictability of intraorally welded titanium bar in immediate loading implants.

## 1. Introduction

The technique of welding portions of intraoral prosthesis has demonstrated many advantages in clinical practise. In fact, the procedure can be performed directly in the mouth eliminating the possibility of errors or distortions due to impressions. In particular, the intraoral welding is very effective in immediate loading of dental implants positioned in atrophic edentulous ridges. So, in case of immediate loading, adequate fixation and stability of implants are very important conditions to prevent the risk of micromovements and loss of implants. In this context, a rigid splinting seems to have an important role in response of peri-implant tissues, since it is able to reduce stress exerted on implants.

The immediate fixation of more implants can be achieved by intraoral welding of abutments. Intraoral welding allows getting immediate retention of osseointegrated implants. The method consists in welding a titanium bar directly to the abutments in the oral cavity before the immediate loading.

The main advantage of intraoral welding consists in placing definitive restoration on the same day of surgery, or few days later. In fact, the lack of stability and retention of denture is the main discomfort complained about by totally edentulous patients.

Degidi and coll [1–8] have published numerous studies about immediate loading of multiple implants by welding a titanium bar directly on abutments in order to create a metal-reinforced temporary or definitive restoration. The results showed that one-piece implant is better than two-piece for intraoral welding.

In 2006 Degidi and coll [1] published a new method defined syncrystallization. This technique consists in splinting multiple implants with a rigid titanium bar welded on abutments. It presents the advantage of immediate restoration on the same day of surgery, stability, and retention of implants in the early stages of bone healing and less implant fractures due to reducing time of restoration [1].

Here we describe a case of atrophic mandible treated with five implants and a welded bar.

## 2. Case Report

**Figure 1:**
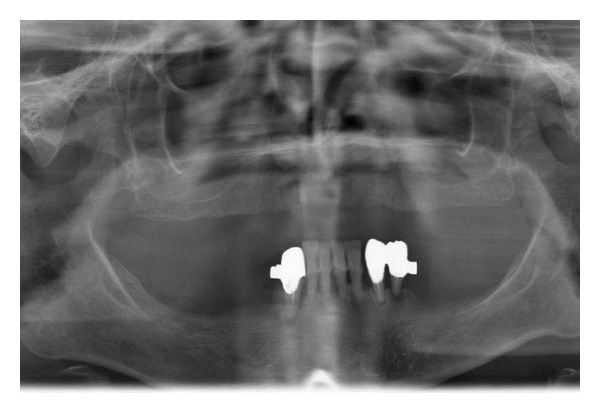
Panoramic X-ray with periodontal teeth.

**Figure 2:**
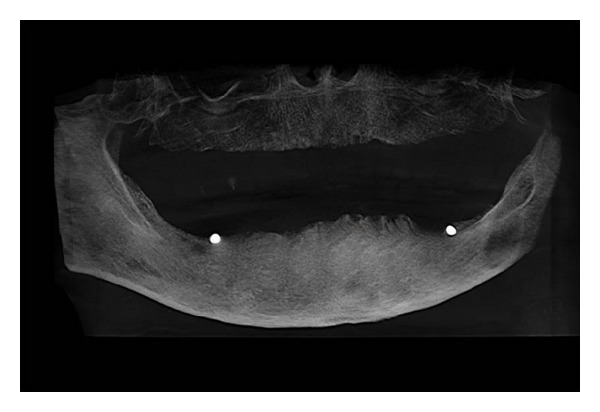
Denture with radiopaque markers used as template.

**Figure 3:**
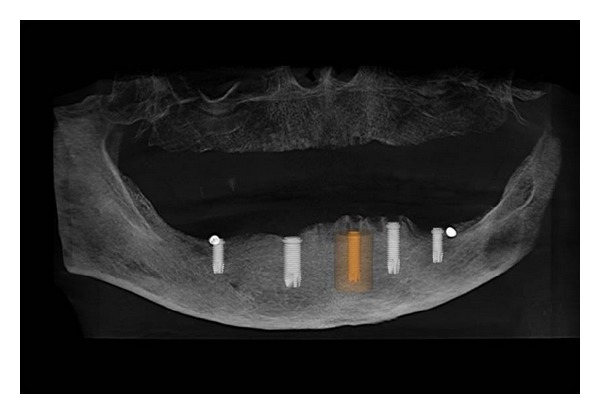
Computer-guided implants technique.

**Figure 4:**
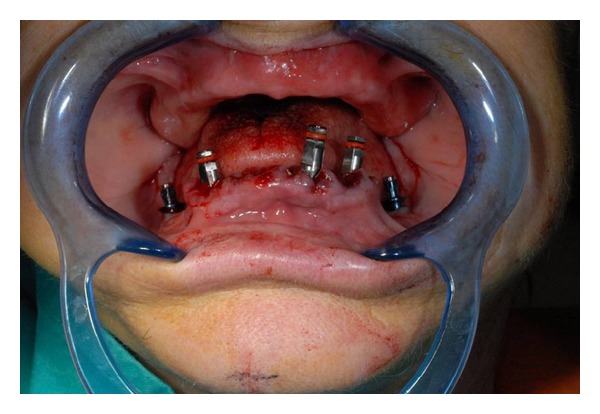
Implants positioned in the mandible.

**Figure 5:**
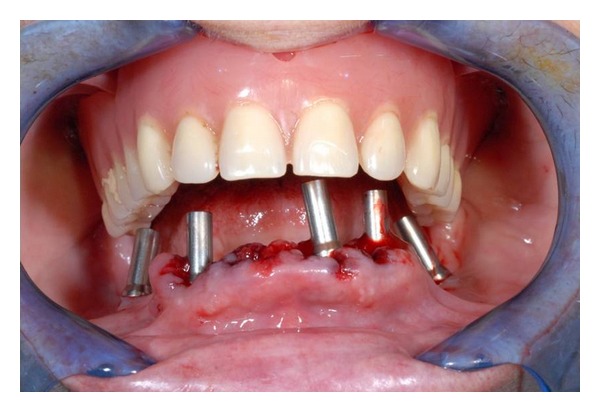
Abutments screwed to implants.

**Figure 6:**
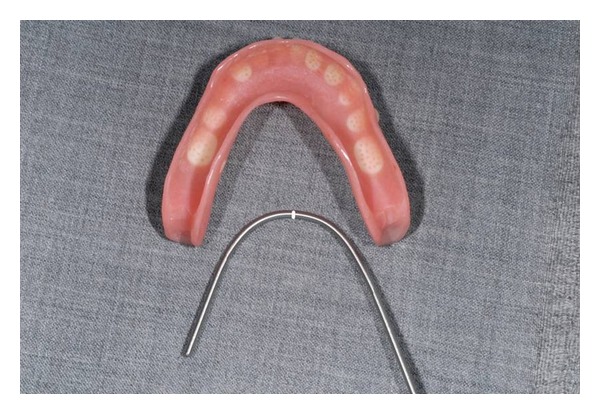
Titanium bar constructed by the dental technician.

**Figure 7:**
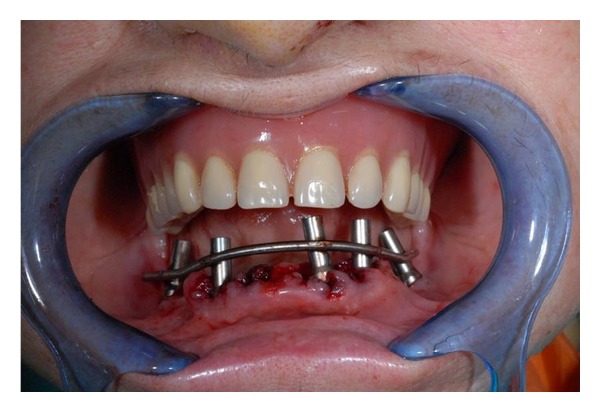
Titanium bar welded on abutments.

**Figure 8:**
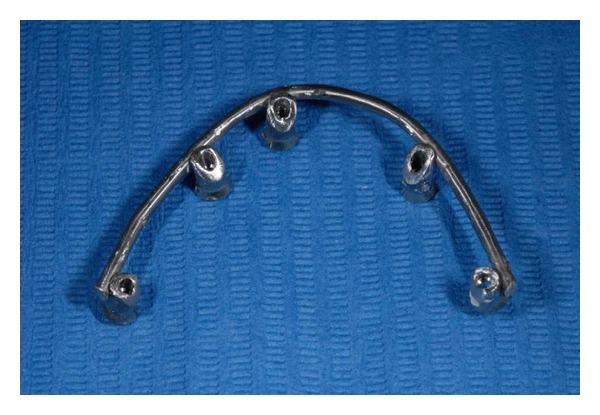
Titanium bar removed from mouth.

**Figure 9:**
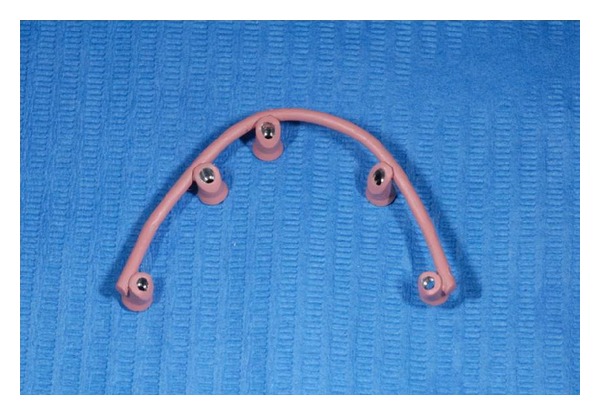
Titanium bar covered with pink opaque.

**Figure 10:**
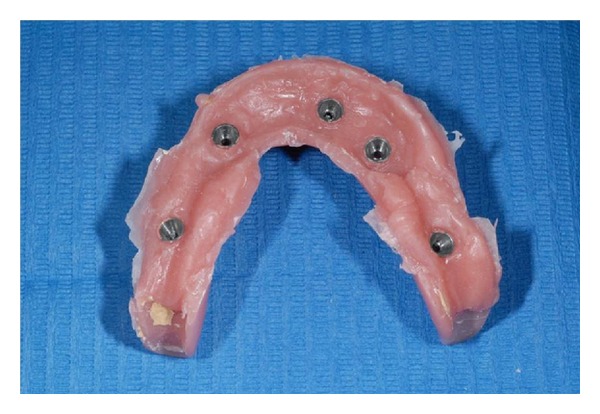
Titanium bar fixed to the denture with acrylic resin.

**Figure 11:**
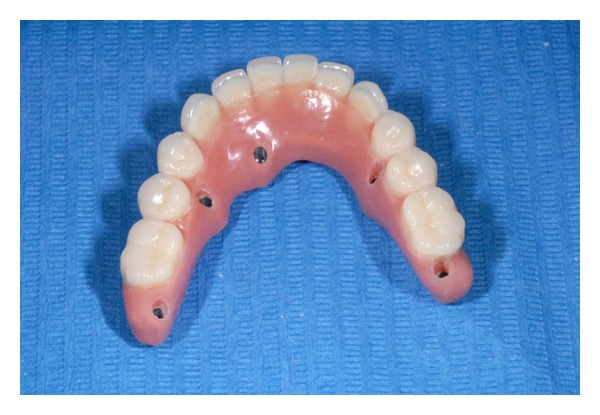
Final prosthesis with drilling for screw tightening.

**Figure 12:**
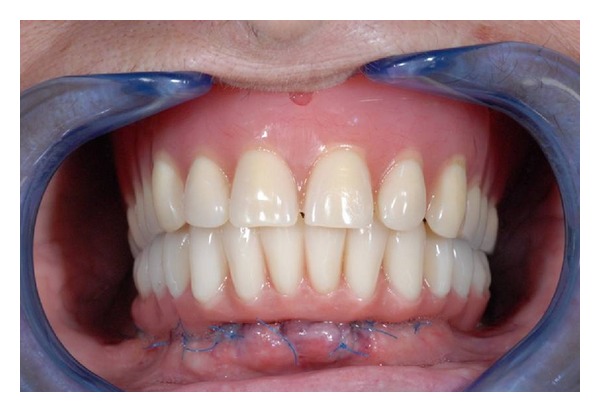
Final restoration.

**Figure 13:**
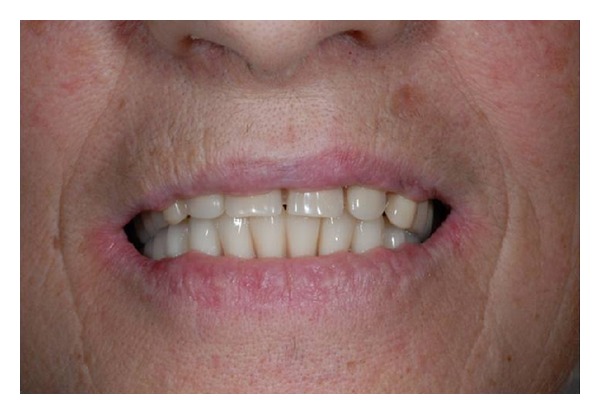
Final restoration.

A 67-year old female came to our clinic for an examination. The medical history did not reveal any systemic diseases and the patient confirmed she did not take any kind of medication. The orthopantomography showed periodontal teeth which subsequently were extracted (Figure 1). Before extraction, an impression was taken to perform a provisional denture. This device was not stable and she had a great discomfort in speaking and eating. It was performed a ConeBeam (Sirona ORTHOPHOS XG) to study the anatomy of the mandible in relation to the position of the inferior alveolar nerve. Based on the data collected we decided to place five implants to stabilize her denture. Two radiopaque markers were inserted in the denture (Figure 2) used as template. Then the patient was undermitted to another panoramic X-ray, wearing the dentures with markers, and on this basis, a computer guided implantology surgery was programmed with the appropriate software (Figure 3). Subsequently the implants were placed in the mandible in a computer guided way. The anaesthesia of the mandibula was obtained by the injection of articaine. Five implants (Dentalplanet Modena, Italy) were inserted, preparing sites with continuous physiological solution irrigation. After the surgical procedure (Figure 4), five abutments with but joint connection were screwed to the implants (Figure 5). Then, a bar previously constructed by the dental technician (Figure 6) was welded intraorally (welding produced by Implamed srl. Cremona, Italy) (Figure 7) in order to fix the position. The bar was removed from the mouth with the abutments (Figure 8) and covered with pink opaque film (Figure 9). The prosthesis was then connected to the bar with acrylic resin (Figure 10) and tightening screws to abutments (Figure 11). The patient was checked after two days and fifteen days and during this period no problems were noted (Figures 12 and 13).

**Figure 14:**
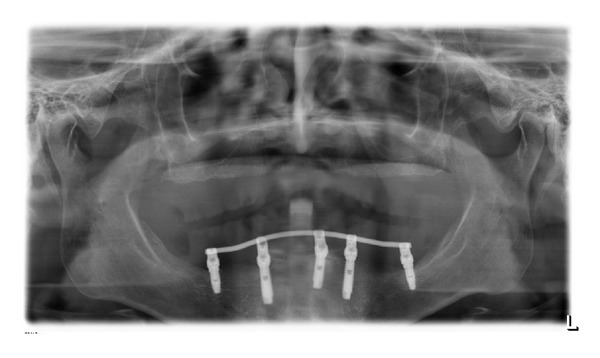
Panoramic X-ray after one-year follow-up.

Patient was very satisfied and no adverse effect was detected in the subsequent 1 year follow-up (Figure 14).

## 3. Discussion

The lack of stability and retention is responsible for oral complains associated with dentures. Besides, sometime implant rehabilitation of atrophic mandible may be impossible because of insufficient bone. Immediate loading implants with definitive denture have demonstrated long-term success and high predictability. Other studies confirm successful oral rehabilitation of edentulous atrophic maxilla with a fixed, definitive restoration supported by an intraorally welded titanium bar on the same day of implant placement surgery [2, 3].

Avvanzo demonstrated that dental abutments, intraorally welded with a titanium bar, allow immediate loading implants and provisional or definitive restoration during healing of bone, without problems of micromovements and implants loss. Immediate denture improves patient's compliance due to a more comfortable prosthesis [9].

Immediate loading of implants may be successfully achieved with intraoral welding technique when they are positioned in zygomatic bone too. In fact it is possible to successfully rehabilitate the edentulous atrophic maxilla with a permanently fixed prosthesis supported by an intraorally welded titanium framework attached to standard and zygomatic implants on the day of surgery. They show stability and prosthetic success rate at the 12-month [4] and 3-year follow-up [5, 6]. The intraoral welding technique seems to have no adverse effect on marginal bone loss and implant survival too [7]. Successful oral rehabilitation has been demonstrated in the edentulous mandible using SynCone 5-degree abutments for an immediate and definitive restoration supported by an intraorally welded titanium bar [8].

Another method to connect the abutments to the bars is represented by the laser welding. Kuo et al. in 2006 described a new technology for immediate loading implants using laser welded bars applicable to various implant systems and clinical situations [10].

In today's dental literature, most frequently, esthetics are addressed with fixed restorations. This technique gives the opportunity to provide patients with very good esthetic outcomes with a hopeless dentition utilizing dental implants, laser-welded titanium components, and characterized acrylic resin prostheses. The definitive prostheses provide excellent facial support, phonetics, esthetics, smile line, and function. Laser-welded titanium frameworks offer many advantages for the patient, clinician, and dental technician [11]. Supporting the data that laser welding is safe for patients and implant survival, Fornaini et al. published a case report suggesting that there are no side effects in using intraorally laser welding technique [12].

A study by Silva investigated the influence of laser welding and electroerosion procedure on the passive fit of fixed implant-supported titanium frameworks, concluding that frameworks may show a more precise adaptation if they are sectioned and laser welded. In the same way, electroerosion improves the precision in the framework adaptation [13, 14].

De Aguiar in his study compared the accuracy of fit of three types of implant-supported frameworks cast in Ni-Cr alloy: specifically, a framework cast as one piece compared to frameworks cast separately in sections to the transverse or the diagonal axis and later laser welded. Results of this study showed that casting diagonally sectioned frameworks lowers misfit levels of prosthetic implant-supported frameworks and also improves the levels of passivity to the same frameworks when compared to structures cast as one piece [15].

Lack of passivity has been associated with biomechanical problems in implant-supported prosthesis. De Castro evaluated the passivity of three techniques to fabricate an implant framework from a Co-Cr alloy by photoelasticity. It was concluded that there were no differences in forces exerted on implants [16].

## 4. Conclusion

Rigid splinting of multiple implants with intraoral welding technique results in a predictable fixation in the early stage for bone healing with a significant reduction of the micromovement problem and implants loss.
